# An education resource for human papillomavirus oropharyngeal cancer patients: think-aloud interviews

**DOI:** 10.1007/s00520-023-07592-y

**Published:** 2023-02-11

**Authors:** Ashleigh R. Sharman, Eliza M. Ferguson, Haryana M. Dhillon, Paula Macleod, Julie McCrossin, Puma Sundaresan, Jonathan R. Clark, Megan A. Smith, Rachael H. Dodd

**Affiliations:** 1 Sydney School of Public Health, Faculty of Medicine and Health, The University of Sydney, Room 127A, Edward Ford Building A27, Sydney, NSW 2006 Australia; 2 Centre for Medical Psychology & Evidence-based Decision-making, School of Psychology, Faculty of Science, The University of Sydney, Sydney, Australia; 3 Psycho-Oncology Cooperative Research Group, School of Psychology, Faculty of Science, The University of Sydney, Sydney, Australia; 4 Northern Sydney Cancer Centre, Royal North Shore Hospital, Sydney, New South Wales Australia; 5 Cancer Voices, Cancer Voices South Australia, Adelaide, Australia; 6 Radiation Oncology Network, Western Sydney Local Health District, Sydney, New South Wales Australia; 7 Sydney Medical School, Faculty of Medicine and Health, The University of Sydney, Sydney, Australia; 8 Department of Head and Neck Surgery, Chris O’Brien Lifehouse, Sydney, New South Wales Australia; 9 The Daffodil Centre, a joint venture between Cancer Council NSW and The University of Sydney, Sydney, New South Wales Australia; 10 Sydney Health Literacy Lab, The University of Sydney, Sydney, Australia

**Keywords:** Communication, Head and neck cancer, Human papillomavirus, Oropharyngeal cancer

## Abstract

**Purpose:**

The human papillomavirus (HPV) is well recognised as a factor in developing oropharyngeal cancer (OPC). A booklet for HPV-OPC patients aimed to deliver evidence-based messages in everyday language, in a way to minimise negative psychological impacts on patients. Our study explored the suitability of the booklet for use.

**Methods:**

Participants were recruited through social media and interviewed via Zoom. Participants were shown the booklet and a think-aloud method elicited real-time reactions to the content. Responses were analysed for each section and coded as either for or against for content, with other responses thematically analysed using NVivo.

**Results:**

The sample comprised 24 participants: patients (*n* = 19) who completed treatment for HPV-OPC and partners of survivors of HPV-OPC (*n* = 5). All participants found the booklet useful, and most wished the resource had been available previously. Some indicated the information was new to them. The majority agreed the booklet would be best delivered by their specialist at point of diagnosis and would be a useful resource for friends and family. Most participants gave feedback on improvements to the booklet in terms of comprehension and design. Overall, participants found the content easy to understand. Most participants found that it helped to reduce shame and stigma associated with HPV as a sexually transmitted infection.

**Conclusion:**

An evidence-based booklet for HPV-OPC patients and their partners is acceptable. Implementation may be feasible in routine clinical practice, specifically at time of diagnosis. Adapting the content will help optimise the efficacy of the booklet in facilitating communication between all stakeholders.

## Introduction

Causal factors for head and neck cancer (HNC) have traditionally been tobacco and alcohol consumption [1]. More recently, the human papillomavirus (HPV) has been recognised as playing a larger causal role in oropharyngeal cancer (OPC), as tobacco control has reduced smoking-related OPC [2, 3]. In Australia, HPV-related OPC has increased from 20.2 (1987–1995) to 63.5% (2006–2010) of all OPC [4].

HPV is a common sexually transmitted virus which can be transmitted via intimate skin-to-skin and mucosal contact. There is limited understanding among patients that HPV infection is extremely common, is a result of usual sexual behaviour, and does not indicate that their partner has been unfaithful [5–7]. Many people with HPV-OPC report shame and stigma associated with their diagnosis, adding distress for patients and partners [8]. However, a study examining awareness that HPV is a very common infection among female students has been associated with reduced shame and stigma [9]. Hence, it is vital that health professionals are prepared to discuss HPV, its sexually transmitted nature, and its high prevalence with OPC patients.

Health professionals are reported to lack confidence in discussing HPV with patients [10]. The changing demographic of patients with OPC presents novel challenges to health professionals regarding the types of questions posed by patients and their priorities. Specifically, patients with HPV-OPC tend to be from more recent birth cohorts [4] and want to be more informed [5]. Despite the increasing prevalence of HPV-OPC, there is a lack of information available to assist patients and their families in understanding HPV and its role in OPC. Health professionals have expressed the need for a clear plan of what to tell patients about HPV and have supported the concept of an information booklet for use with patients [10]. Moreover, previous research on HPV and cervical cancer found provision of key information reduced the negative psychological impact on patients such as shame and stigma [7, 9]. There is a need for high-quality, evidence-based information to optimise communication and assist the conversation between health professionals and patients.

We designed an evidence-based booklet for HPV-OPC patients, informed by interviews with health professionals, and patients and their partners [10]. The booklet was designed to support health professionals in communicating the role of HPV in OPC, answer patients’ most frequently asked questions in clear and concise everyday language, and minimise negative psychological effects on the patient. This study aims to measure readability, comprehensibility, and acceptability of the booklet to patients and their partners through think-aloud interviews.

## Methods

### Study design

The “think-aloud” method was used in this study to elicit real-time responses to the booklet content. Participants were asked to vocalise their inner thoughts and/or feelings while reading through each section of the booklet. This method allowed access to higher order cognitive processes where introspective thought was collected as verbal data [11]. This technique has previously been used in colorectal cancer information resources [12] and web-based information tools for people with cancer [13].

### Booklet development

The booklet was developed as part of a submitted PhD thesis, completed in 2016 (RD). Qualitative interviews with health professionals, HPV-OSCC patients and their partners, informed creation of the booklet content alongside evidence-based practice. Once drafted, the booklet underwent grammar and readability checks. Subsequently, the booklet was reviewed by clinical experts and patients. The booklet’s graphic design was completed by a social marketing company and behavioural insight team with experience in health-related projects.

### Participant recruitment

The study was advertised on social media (Twitter, Facebook) of head and neck cancer associations and support groups/persons. While there was no pre-determined sample size, the study sought to recruit around 25 participants without purposive sampling against any demographic criteria. Individuals expressed interest in participating by responding to the study advertisement and providing their e-mail details through online data capture using Qualtrics software (Version 2021). Individuals were eligible to participate if they were 18 years or older and they, or their partner, had been diagnosed with HPV-OPC. Eligible participants were emailed an information sheet, consent form, and pre-interview questionnaire. Study data were collected and managed using REDCap (Version 11.2), an electronic data capture tool hosted at The University of Sydney. Participant interviews took place in January and February 2021.

### Study procedure

Prior to the interview, participants completed a short demographic questionnaire (e.g., age, sex, education level, diagnosis stage) with responses stored via REDCap. Health literacy was measured using the Single Item Literacy Screener which asked, ‘How often do you need to have someone help you when you read instructions, pamphlets, or other written material from your doctor or pharmacy?’ (never/rarely/sometimes/often/always) [14]. The “think-aloud” method was explained to participants, and they practised using an unrelated control booklet. Participants were then shown the HPV-OPC educational booklet and asked to read the content and pause to give their thoughts whenever a red dot was reached (generally at the end of every sentence). Participants were also asked a series of true/false comprehension statements at the end of the booklet. Interviews were conducted by two members of the research team (EF, HD) virtually via Zoom (Zoom Video Communications Inc., 2016). The interviews were audio-recorded and transcribed verbatim. Data saturation was reached when no new themes had emerged from three consecutive interviews. Data collection ceased at this point [15].

### Data analysis

Interview recordings were audio transcribed using TRINT (Trint Ltd., 2021) and quality assured (by AS). Deidentified responses were analysed for each section of the booklet. A selection of transcripts was read by four members of the research team (AS, PM, HD, RD) to identify themes and cross-check codes. These were developed into a coding framework (by AS, RD) and data coded into common themes using NVivo qualitative software (QSR International, Version 12) (by AS). This process, known as thematic analysis [16], aims to identify a set of main themes capturing the views and feelings of participants, represented by quotations. Participant reactions to specific sections of the booklet content were also coded as either for the content, against the content, or recommending changes to the content.

## Results

Forty-six people expressed interest in taking part, of which 26 consented and 24 were interviewed. Of the two participants who consented but did not take part, one participant forgot about the interview and could not be rescheduled; another was found to be ineligible. The remaining 20 participants who expressed interest in the study, but did not return a participant consent form, did not give a reason for doing so.

There were 19 patients diagnosed with HPV-OPC and five partners or friends of survivors with HPV-OPC who participated. Interviews lasted on average 83 min (range 55 to 140). Participant demographics are reported in Table 1, with most participants completing education beyond high school (79%), and all had adequate health literacy. Most patients were diagnosed between 2015 and 2019 (53%), with 63% advised of the association of HPV with their own cancer at time of diagnosis (see Table 2). Two participants did not complete the pre-interview questionnaire.

**Table 1:** Participant demographics

Sample (*n* = 24)		*n* (%)
	Female	13 (54%)
	Male	11 (46%)
Age^a^		55 (median, SD = 8.8)
Country of birth^a^	Australia	17 (71%)
	United Kingdom	5 (21%)
Aboriginal and Torres Strait Islander^a^	No	22 (92%)
Education^a^	University degree	12 (50%)
	Diploma or certificate	7 (29)
	High school (year 12)	1 (4%)
	High school (year 10)	1 (4%)
Relationship^a^	Single	3 (13%)
	In a relationship	19 (79%)
Employment^a^	Full-time	10 (42%)
	Part-time	4 (17)
	Self-employed	1 (4%)
	Recovery leave	1 (4%)
	Retired	6 (25%)
Help needed with medical information^a^	Never	20 (83%)
	Rarely	2 (8%)

^a^Completed total (*n* = 22)

**Table 2:** Participant diagnosis details+

Variable	Patients (*n* = 19)	Partners (*n* = 5)^b^
Age at diagnosis^a^	54.4 (mean)	(NA)
Diagnosis year^a^		
2005–2009	1 (5%)	0
2010–2014	5 (26%)	0
2015–2019	10 (53%)	3 (60%)
2020–2021	2 (11%)	1 (20%)
Diagnosis stage^a^		
Stage 1	2 (11%)	1 (20%)
Stage 2	4 (21%)	2 (40%)
Stage 3	6 (32%)	1 (20%)
Stage 4	4 (21%)	0
Don’t know/can’t remember	2 (11%)	0
Time advised of HPV association with their cancer^a^		
At time of diagnosis	12 (63%)	4 (80%)
After diagnosis	5 (26%)	0
Other (many years later)	1 (5%)	0

^a^1 patient data missing, 1 partner data missing

^b^Partners reflect details of the patients’ cancer diagnosis

### Booklet response

**Fig. 1:**
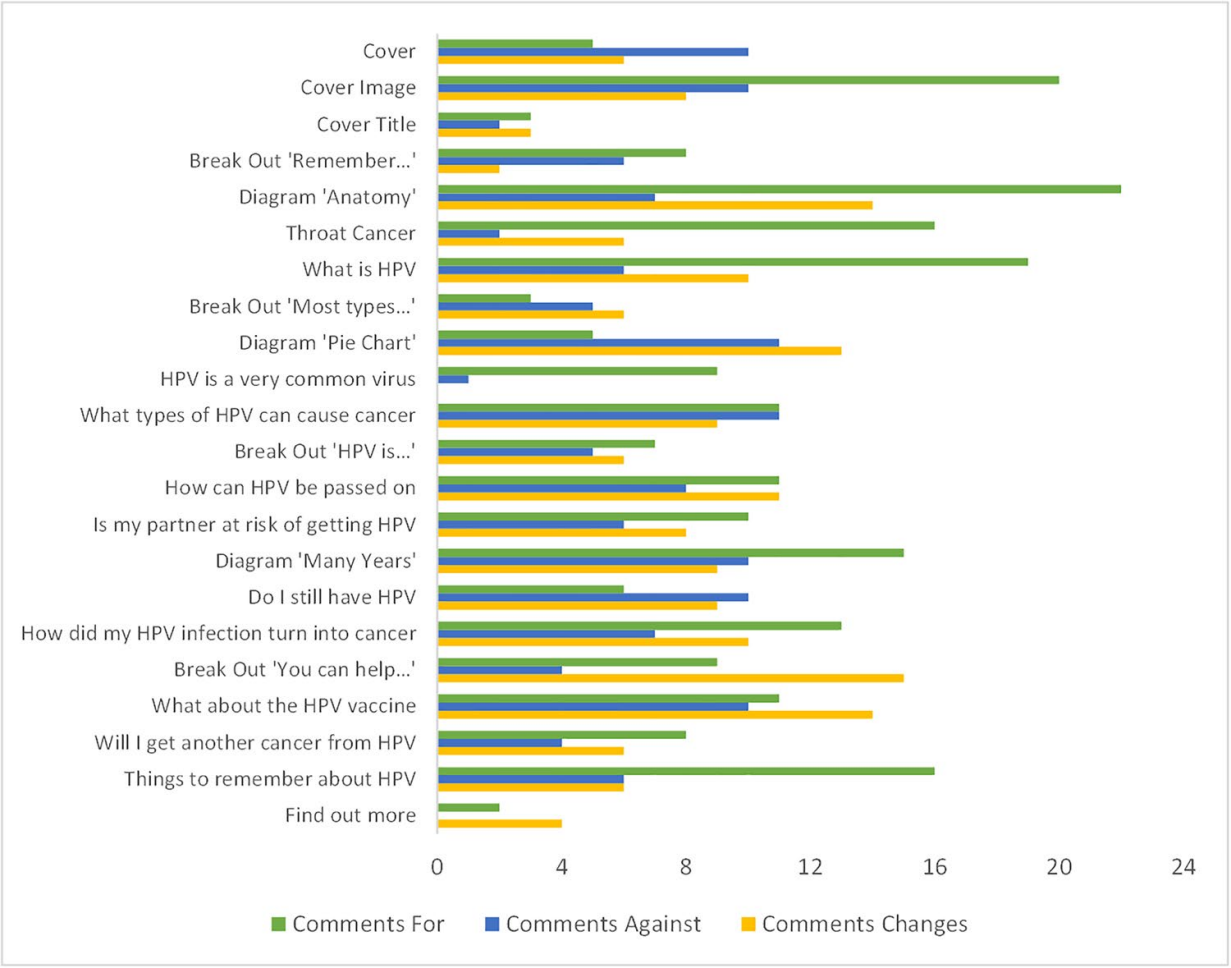
Number of comments for, against and recommended changes to the booklet content, received from participants (*n* = 24) by booklet section

Participants made positive and negative comments about the booklet content, also recommending changes, at various marked points in the booklet as shown in Fig. 1.

The sections most frequently commented on were the cover image and a diagram depicting where throat cancer can occur. The least frequently commented on sections were the cover title and ‘find out more’ section.

**Fig. 2:**
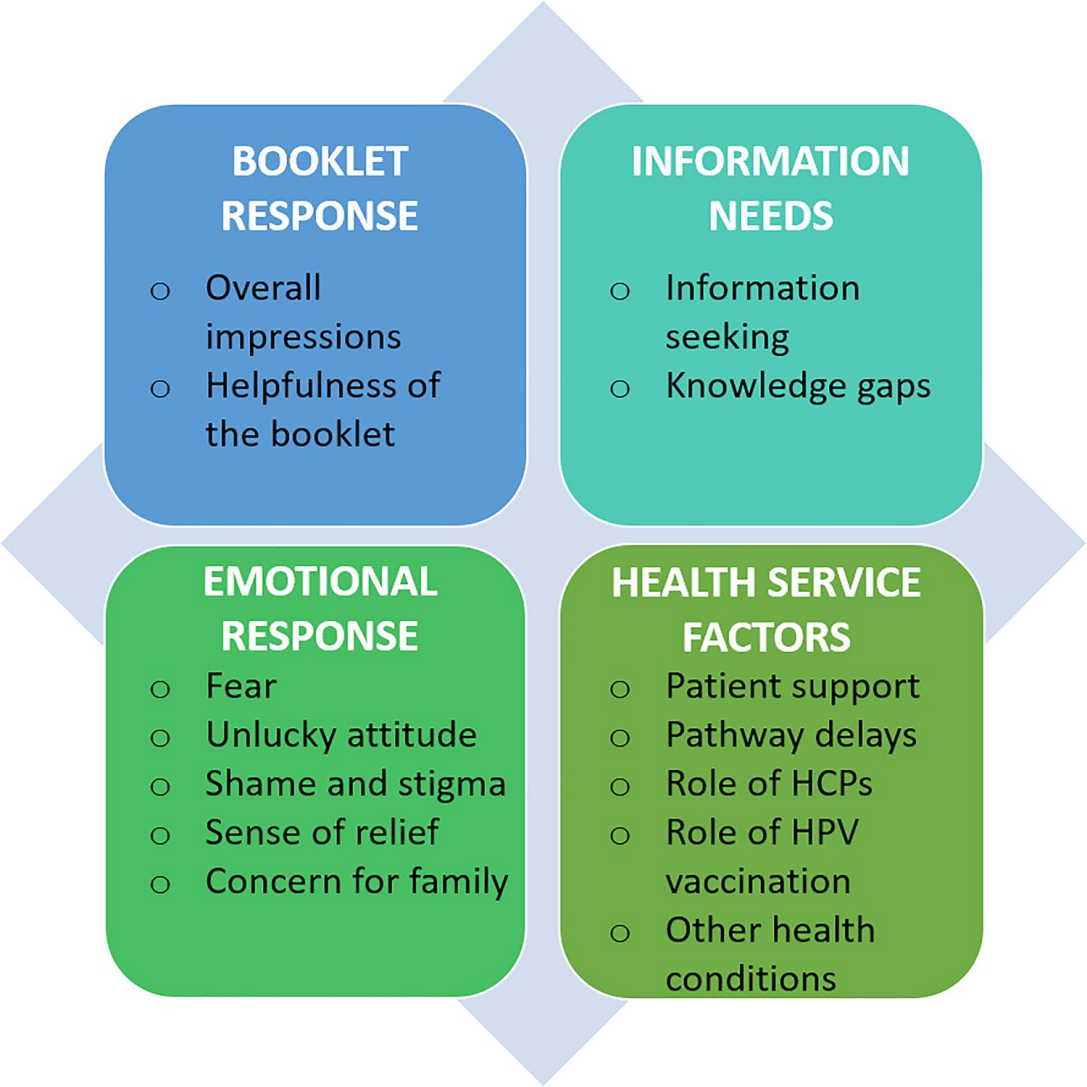
Themes and sub-themes from qualitative interviews

The think-aloud method also elicited responses to the booklet that related to experiences as an HPV-OPC patient, or partner/friend of a patient, with quotes (Table 3) given to support the themes and sub-themes in Fig. 2.

**Table 3:** Quotes from interviews to support theme

Code	Theme	Sub-theme	Representative quote
Q1	Booklet response	Overall impressions	“I love the plain language and so far, I'm really pleased with the way you're saying things in Common English, I haven't come across really any jargon, so that's a plus” (P018 – patient)
Q2			“I understand it all, I was just more pausing from what people potentially who, you know, actually thinking about inclusion and diversity. And you're going to have different genders. You're going to have different age ranges, and you're going to have a diversity of cultures and languages, English being first or second language. I think your biggest challenge is because we are a multicultural society” (P005 – patient)
Q3		Helpfulness of the booklet	“Because family and everyone want to know, and you can just hand to them. And they can see for themselves what it is. And you don't have to explain. It would've been good” (P003 – patient).
Q4			“I think there's a lot of places that you could circulate that, through the college of GPs, through the dental schools to all the dentists and they only need a PDF…for specialists, I think they need to have that resource all sent to them as soon as you can” (P015_P – partner)
Q5	Information needs	Information seeking	“I guess I'm looking at it kind of in hindsight, I suppose, and trying to have it as clear as possible, knowing that when you read things and you're in a little bit of shock, you want to be as clear as possible” (P009 – patient)
Q6			“Because I went on to the Internet and looked up a lot of stuff… And that sort of booklet just puts it into the realm of reality rather than all the stuff you read on the Internet” (P006 – patient)
Q7			“I think my experience is a lot of people want that detail when they're going through their diagnosis and treatment plan. I just don't like uncertainty” (P007 – patient)
Q8		Knowledge gaps	“There's an awareness, obviously, of head and neck cancers and throat cancer, but the fact that it can be triggered by HPV was news to me” (P004 – patient)
Q9			“So, I have kind of had an understanding of what it is. But how I got it, whether I could pass it on, that definitely were two questions that came to mind when I was first diagnosed” (P005 – patient)
Q10			“I think you don't only get HPV from sex though. That's my understanding. I understand you can get it from kissing or yeah, it's not just sexual relations” (P011 – patient)
Q11	Emotional response	Concern for family/partner	“When I was diagnosed with it and they went through the whole treatment and then my thoughts went to protection of our children, can our children be protected?” (P001 – patient)
Q12			“Obviously, I'm going to tell my partner, but I didn’t think, my concern was not that it was going to cause him cancer. It was more the fact that he was going to get HPV” (P005 – patient)
Q13		Fear	“Does that mean I can go through all of this treatment and then it comes back? Like this generates a few questions so that uncertainty around that is a little bit scary. My concern probably wasn't whether or not HPV would lead to another cancer it's just whether or not this cancer would come back or be treatable” (P007 – patient)
Q14			“You know what the web is like, you never go ‘HPV and cancer’ and then see what's on the web. And I'm like, oh my God! Yeah, that's that kind of, I left the computer feeling pretty shaken…” (P018 – patient)
Q15			“I think this was almost the unasked, unspoken question for a couple of weeks, I've got to say [what if I pass it on?]” (P020_P – partner)
Q16		Sense of relief/comfort	“That's important to mention, just so people know there was nothing they could have done… I think that'll give people a little bit of comfort in a way if they've already been diagnosed and just again, it was probably nothing they could have done to prevent it” (P003 – patient)
Q17			“I like the word common because I feel that everyone knows that it's not from risky behaviour... That most humans, regardless of whether they're heterosexual, gay, trans, whatever it happens to be, are at risk of getting this cancer that it's not, it's not a certain group of people that are at a higher risk than others” (P020_P – partner)
Q18			“You’ve also been told you won the lottery because it responds better to treatment. I didn't know about HPV …that it responds to treatment better… Gives me hope that this responds better than anything else” (P004 – patient)
Q19		Shame and stigma	“But everyone wants to know how you got it and you know; it's got a sexual connotation to it. That's still a little bit embarrassing” (P013 – patient)
Q20			“Well, I've been with the same person since I was 18 years old. So, I'm not promiscuous. What does it matter how many partners you have? You can get it from just one person? What does it matter, risk going up with a number of partners, what, how is that helpful? Judgmental” (P011 – patient)
Q21			“Yeah, because presumably I could have been [diagnosed with HPV] p16 and [also be] a smoker and a drinker, so yeah that might be, stigmatising might be a bit strong, but yeah, I'm just not sure of that” (P022 – patient)
Q22		Unlucky attitude	“I'm just unlucky and I can deal with unlucky… Rather than something that I was to blame for” (P004 – patient)
Q23			“That's like a bit of a bummer, isn't it? That all of us reading this, we're in the one percent” (P012 – patient)
Q24			“OK, so I'm one of the very few people that turn into throat cancer. Again, it's kind of like... I guess I want the explanation for it” (P009 – patient)
Q25	Health service factors	Patient support	“Somebody our age, from a psychological point of view, having support, living on your own would be very hard. Doing it with a partner is hard enough on the partner… Having supportive friends, it’s critical, supporting partners, family (P008 – patient)
Q26			“You know, for me the one big thing I found was the support group, the reason for that was a bunch of people who all had not the same problem, but similar things or had answers that I didn't have” (P008 – patient)
Q27			“To help the next person. This why I'm doing it, not basically for myself, but for the next person” (P001 – patient)
Q28		Other medical conditions	“I'm not seeing the word herpes. And I have herpes and HPV in my head as for what HPV means so cold sores, genital herpes, that sort of thing” (P004 – patient)
Q29			“I don't know that people who are first diagnosed - it's pretty stressful - are fully aware of how to live with the side effects, because my life's been forever changed and I, you know, have various disabilities now” (P011 – patient)
Q30		Pathway delays	“So, it's just making people aware if you get a sore throat that goes on longer than a few weeks, and it's not resolved with a course of antibiotics, don't shop around, don’t go to five GP's. Demand a referral to an ENT really quickly. Just delays the diagnosis, which means your prognosis is worse and worse” (P015_P – partner)
Q31		Role of healthcare professionals	“It's good to actually emphasise that because I think some people might think, and I know my partner said actually I think once or twice, I don't really need to go to the dentist. I was like, yes you do!” (P002_P – partner)
Q32			“I think they need to emphasise the fact that the dentist can play an important part in this… I think the dentistry mob should be really inducted into the system well and truly” (P006 – patient)
Q33		Role of HPV vaccination	“I think that's great that they did the vaccine, and I hope that it really does show the numbers, reduce numbers of throat cancer because of it” (P036 – patient)
Q34			“And I think, OK, now what about the HPV vaccine? That it won't help currently, but it will help prevent further infections. So, does that mean that I can still have the vaccine?” (P009 – patient)
Q35			“And that I guess that's my question, for your partner and, but I guess that's something, you read that, and you go, so does my partner. Should she now go and get a vaccination?” (P046 – patient)

#### Overall impressions

Most participants thought the booklet was clear and easy to read (Q1). Several participants wanted to ensure the booklet was accessible to Aboriginal and Torres Strait Islanders and culturally and linguistically diverse communities (Q2). Other feedbacks are related to additional content, such as inclusion of sections such as mental health and wellbeing, treatment options, and side effects.

#### Helpfulness of booklet

Participants overwhelmingly supported the booklet as a patient resource. Citing a lack of physical resources, participants expressed a preference to receive the booklet at diagnosis and viewed it as a resource also for family and friends (Q3). Participants commented positively on the booklet’s simple information and key messaging via break-out boxes, suggesting it could have a broader application as an education tool for healthcare professionals (Q4).

### Information needs

Participants reflected on information needs and knowledge gaps from when they were diagnosed.

#### Information seeking

Participants frequently expressed feeling overwhelmed with information at diagnosis (Q5) and that the booklet would have provided a point of focus and something to refer back to. While some participants discussed the feeling of information overload, many also sought information online (Q6). Some participants expressed a desire for more detail (Q7), in particular statistics from credible sources.

#### Knowledge gaps

Prior to their diagnosis, many participants stated they were unaware of the link between HPV and OPC (Q8). Participants frequently expressed confusion around the sexual nature of HPV transmission (Q9). They were also uncertain if HPV could be caught if they had never had sex or through kissing (Q10).

### Emotional response

Participants expressed both positive and negative emotions while reading the booklet. Specifically, patients shared how a diagnosis of HPV-OPC made them feel, how their emotional needs were looked after, and how the booklet helped to address these feelings.

#### Concern for family/partner

Upon diagnosis, some participants acknowledged their concern for those closest to them (Q11). Others expressed concern for their partner or those they were in a sexual relationship with (Q12).

#### Fear

Most participants worried about recurrence of their cancer after completion of treatment (Q13). This worry was supported by some participants’ lived experience of cancer, and that both receiving a cancer diagnosis and using the Internet as an information source are scary (Q14). Some participants also worried that their HPV infection might come back and they would unwittingly pass on the virus (Q15).

#### Sense of relief/comfort

Participants frequently commented that the booklet’s content provided a sense of relief. Many were comforted to know there was nothing they could have done to prevent HPV turning into cancer (Q16). Other participants took comfort in how common the HPV virus is in the general population, yet rarely develops into cancer (Q17). Many participants also found the better prognosis in HPV-OPC hopeful and encouraging (Q18).

#### Shame and stigma

Many participants were reminded of the embarrassment they felt following their HPV-OPC diagnosis (Q19), contributing to feelings of self-blame and guilt. However, several participants also remarked that some of the booklet’s content was confrontational and judgemental (Q20). A few participants also drew attention to the stigma surrounding smoking and drinking as problematic within the booklet (Q21).

#### Unlucky attitude

The majority of participants explained a diagnosis of HPV-related cancer as unlucky (Q22). This attitude stemmed largely from the understanding that while HPV infection is common, a related cancer is rare (Q23). However, it also made some participants express feelings of helplessness, asking why they were part of the “unlucky” 1% (Q24).

### Health service factors and general feedback

These themes reflect participants’ experiences of medical services and treatments, or information arising during the “think aloud” process, that were not directly related to the booklet.

#### Patient support

A selection of participants cited the role of faith, HPV diagnosed celebrities, family, and friends as crucial supports through treatment (Q25). Medical staff were commended for their services; however, some participants found head and neck cancer support groups and associations most helpful (Q26). Of these participants, many were also actively involved in support groups and associations, where giving back to the head and neck cancer community was a key reason for joining this study (Q27).

#### Co-morbidities

A handful of participants raised other medical conditions, questioning if they were related to their HPV-related cancer. These included auto-immune disorders, neuropathies, urinary tract infections, and herpes (Q28). Other participants remarked on the effects of treatment leading to current medical conditions (Q29).

#### Pathway delays

The most frequently commented on pathway delay was diagnostic. Several participants reported there was a significant delay from GP visits to accessing a specialist referral where a diagnosis of HPV-related cancer could be confirmed (Q30).

#### Role of healthcare professionals

Most participants expressed a positive attitude toward healthcare professionals in whom they placed their trust, in particular, the role of GPs, specialist surgeons, nurses, and dentists. The importance of dentists was highlighted due to their role spanning the spectrum of diagnosis to follow-up care (Q31, Q32).

#### Role of HPV Vaccination

Participants overwhelmingly expressed support for the school-based HPV vaccination program (Q33). While participants raised queries over the age at which school children were vaccinated against HPV and expressed confusion over the cost of this vaccination, the greatest concern was whether they, themselves, should have the HPV vaccine (Q34)*.* This concern regarding HPV vaccination also extended to partners and family members (Q35).

### Comprehension statements, quantitative

A total of 16 participants (67%) scored 100% in the true/false comprehension statements at the end of the interview. The remaining eight participants (33%) scored 80%. The two questions most frequently answered incorrectly were: “The HPV infection has some obvious symptoms” (12% incorrectly answered “true”), and “It takes a long time, at least 10 years for HPV to progress to cancer” (17% incorrectly answered “false”) (see Supplementary Materials 1). Incorrect answers were Q3, where participants confused cancer symptoms for HPV symptoms, and Q4, where participants only read this information once in the summary section of the booklet and felt it should be reinforced throughout.

## Discussion and conclusion

### Discussion

Our study demonstrates overwhelming support for this booklet as a much-needed resource for patients and their partners. In giving patients support and reassurance, the booklet’s easy to read content helps to remove shame and stigma often felt due to HPV’s sexual transmission [8]. Preferred to be given at diagnosis by a healthcare professional, the booklet is also a useful resource for family and friends, further validating its potential as an information tool that can be used by health professionals in communicating the role of HPV in OPC. These findings are potentially applicable in the other high-income countries experiencing similarly rising incidence of HPV-related OPC.

Participants were supportive of the booklet’s content, most frequently recommending changes to some of the images and diagrams used. Visual information within health communication is used partly to attract and retain the attention of the reader [17] and in cancer communication, commonly represents people, anatomical images, or visual data [18]. In a study using medical illustrations of pancreatic and cervical cancers, researchers found the preferred detail in illustrations varied with gender and age [19]. This finding needs to be considered if the booklet requires adaptation for a different age group.

Where participants were happy with the amount of content, they often expressed the need to search for additional information online due to a lack of existing resources. This has been established as a common behaviour where cancer patients may prefer to receive resources from a healthcare professional but still require additional detailed information [20, 21]. However, online information seeking often brought with it feelings of fear and distress. This not only highlights the importance of healthcare professionals providing trustworthy and credible resources to patients [22] but also supports the positioning of this booklet online.

Patient education during COVID-19 necessitated both in-person and digital approaches, with digital patient education now increasingly part of standard care [23]. Whether available as a downloadable file, app, e-book, audio book, or standalone website, it is important for evidence-based information to be available in multiple formats [24]. Patient advocacy group websites are also an ideal location to house a wide range of information and support in digital formats [25]. Where information booklets may be considered a low-cost intervention to improve knowledge in cancer patients [26], this booklet may be adapted to other formats and mediums to improve access as areas of future research.

Participants held healthcare professionals in high regard and cited them as an important source of information. The dentist was most commonly highlighted as essential to treatment aftercare, which has been previously reflected in quality-of-life studies of patients with oral cancers [27].

The role of celebrity health disclosure, in the cases of international star Michael Douglas and Australian media personality Julie McCrossin, aided participants in explaining their HPV-related diagnosis to others. A study examining UK press mentions of the link between HPV and head and neck cancer draws attention to the media interest in Michael Douglas’ HPV-related throat cancer diagnosis in 2013 in which he publicly announced oral sex as means of transmission [28]. Despite this, participants felt greater awareness of HPV was still needed with a recent study calling for continued interventions to increase awareness of HPV-related OPC in the general public [29]. Doing so will raise awareness, reduce stigma, and reduce time to diagnosis.

Many participants in this study were supported through diagnosis and treatment by online support groups. A recent study confirmed cancer patients received the greatest benefit from online over offline support groups [30], where participants cited that community, state, and national support groups were their preferred source of evidence-based, trustworthy information. Such organisations may provide essential stakeholder input in ongoing resource development including the development of culturally appropriate materials for Aboriginal and Torres Strait Islander and culturally and linguistically diverse communities.

While the booklet largely helped to bring a sense of relief and comfort, there remained feelings of fear and worry. The fear of cancer recurrence is common in survivors of HPV-related OPC [31]. These feelings remain with participants long after treatment and therefore have the potential to be addressed in information resources [32, 33]. Content suggestions, including mental health and wellbeing, treatment options, and side effects, were also raised by participants. While outside the scope of this booklet, these remain important information needs of patients [5, 6].

The majority of participants were born in Australia, well-educated with high health literacy, working full-time, and in a relationship. While the participant population in this study reflects the typical HPV-related OPC demographic, it does not reflect the full spectrum of people affected. There is a need to examine the utility of this booklet in Aboriginal and Torres Strait Islander, culturally and linguistically diverse, Lesbian Gay Bisexual Transgender Queer, disability, low socio-economic, and low health-literacy groups. Doing so should capture differences across groups to understand content and information delivery needs unique to each group. Our participants were most frequently diagnosed between 2015 and 2019, and had the benefit of hindsight; there is merit in trialling the booklet with patients closer to diagnosis.

This research provides valuable insight into the target patient population’s views of this resource. This study also has the potential to improve communication about sensitive topics such as HPV and minimise the potential of negative psychosocial impact. The results will inform the subsequent roll out of the resource in routine clinical practice, with the findings relevant and applicable across Australia and other countries. We propose to adapt the booklet for use within other cultural groups, involving consultation and translation of the booklet’s content.

### Conclusion

An evidence-based booklet for HPV-related OPC patients and their partners is acceptable to the people in Australia. The booklet was seen as a welcomed educational resource, not only for the patient but also for their friends and family. The booklet also provided a sense of relief and comfort to patients’ understanding of their diagnosis. This has the potential to reduce feelings of shame and stigma associated with HPV as a sexually transmitted infection.

### Practice implications

Implementation may be feasible in routine clinical practice, specifically at time of diagnosis. Responses to the content will help optimise the efficacy of the booklet in facilitating communication between all stakeholders.

## Data Availability

The data that support the findings of this study are available from the corresponding author upon reasonable request.
